# Diagnostic Value of Serum Fibroblast Growth Factor 21 Combined with Fibrosis-4 Index for Hepatic Fibrosis in Metabolic Dysfunction–Associated Steatotic Liver Disease

**DOI:** 10.5152/tjg.2026.25740

**Published:** 2026-06-02

**Authors:** Yang Cao, Xing Zhang, Qian Zhang

**Affiliations:** Department of Infectious Diseases, The Second Affiliated Hospital of Harbin Medical University, Harbin, China

**Keywords:** Fibroblast growth factor 21, fibrosis-4 index, hepatic fibrosis, metabolic dysfunction–associated steatotic liver disease, non-alcoholic fatty liver disease, noninvasive diagnosis

## Abstract

**Background/Aims:**

Metabolic dysfunction–associated steatotic liver disease (MASLD) is a leading cause of chronic liver disease, with significant fibrosis (≥F2) strongly associated with adverse outcomes. The current study evaluated the diagnostic value of serum fibroblast growth factor 21 (FGF-21) combined with the fibrosis-4 index (FIB-4) for the assessment of hepatic fibrosis in patients with MASLD.

**Materials and Methods:**

A total of 125 patients with MASLD were enrolled (91 patients with F0-F1 fibrosis and 34 patients with F2-F4 fibrosis). Independent risk factors for significant fibrosis were identified using multivariate logistic regression. A combined diagnostic model was developed and validated using bootstrap resampling (500 iterations). Diagnostic performance was evaluated using receiver operating characteristic curve analysis.

**Results:**

Compared to the F0-F1 fibrosis group, patients with F2-F4 fibrosis had significantly higher waist circumference (WC), gamma-glutamyl transferase (GGT), homeostasis model assessment of insulin resistance (HOMA-IR), total cholesterol, low-density lipoprotein cholesterol, FIB-4 index, and FGF-21 levels and significantly lower high-density lipoprotein cholesterol (HDL-C) levels. Multivariate analysis identified FIB-4, FGF-21, WC, GGT, and HOMA-IR as independent risk factors, whereas HDL-C was identified as a protective factor. The combined model achieved an area under the curve of 0.806 (95% CI: 0.727-0.886), outperforming FIB-4 (0.743) and FGF-21 (0.735) alone. At the optimal cutoff determined by the Youden index, the combined model maintained comparable specificity while significantly improving sensitivity to 94.12%. The model also demonstrated balanced performance under both specificity-driven and sensitivity-driven strategies.

**Conclusion:**

Combining FGF-21 with FIB-4 improved diagnostic accuracy for significant fibrosis in MASLD, providing a practical, noninvasive tool for early risk assessment and patient stratification.

**Main Points**

- The combination of fibroblast growth factor 21 (FGF-21) and fibrosis-4 index (FIB-4) improves diagnostic accuracy for significant fibrosis (≥F2) in metabolic dysfunction–associated steatotic liver disease (MASLD).
- The combined model achieved a superior area under the curve of 0.806, outperforming either biomarker alone.
- The FGF-21/FIB-4 model demonstrated high sensitivity (94.12%) for detecting significant fibrosis.
- This noninvasive tool offers a practical strategy for early risk stratification in patients with MASLD.

## Introduction

Metabolic dysfunction–associated steatotic liver disease (MASLD), previously known as nonalcoholic fatty liver disease (NAFLD), has emerged as a leading cause of chronic hepatic disorders globally. This disease is closely associated with obesity, insulin resistance, and metabolic syndrome, particularly among patients with type 2 diabetes mellitus.^1^ Since 2020, international multidisciplinary expert consensus has recommended replacing NAFLD with MASLD to better reflect its metabolic basis, whereas nonalcoholic steatohepatitis (NASH) has been renamed metabolic dysfunction–associated steatohepatitis (MASH).^1^^-^^3^ MASLD is characterized by excessive hepatic lipid accumulation in the absence of alcohol consumption and encompasses a disease spectrum ranging from simple steatosis, formerly termed NAFL, to MASH, which may progress to fibrosis, cirrhosis, end-stage liver disease, or hepatocellular carcinoma.^4^^-8^ MASLD has become increasingly prevalent in primary care settings, and the stage of liver fibrosis is a key determinant of long-term outcomes. Progression to the pivotal F2 stage of fibrosis is associated with significantly increased risks of severe complications and mortality. Thus, optimizing early diagnostic strategies for patients with MASLD and fibrosis stage ≥F2 has substantial clinical value.^9^^-^^11^ However, early-stage fibrosis associated with MASLD lacks specific clinical manifestations, and its insidious progression poses major challenges for timely identification of this critical phase. Although F2-F4 fibrosis is associated with poor prognosis, these stages remain partially reversible. Therefore, establishing effective early detection strategies for the pivotal F2 stage is essential to enable timely intervention and improve outcomes.^12^^-^^14^

Liver biopsy remains the gold standard for fibrosis assessment; however, its invasiveness, operator dependence, and sampling variability limit its routine application.^15^^,^^16^ Technological advances have introduced noninvasive alternatives, including vibration-controlled transient elastography, point shear-wave elastography, 2-dimensional shear-wave elastography, magnetic resonance elastography (MRE), and magnetic resonance imaging, and serum-based scoring systems, all of which demonstrate favorable sensitivity and specificity. However, their widespread adoption in primary care remains limited by cost and technical requirements.^10^^,^^17^^-^^19^

The fibrosis-4 index (FIB-4), a noninvasive scoring model based on age, aspartate aminotransferase (AST), alanine aminotransferase (ALT), and platelet count, is widely used for initial screening of significant fibrosis in MASLD and has been endorsed by guidelines from the American Association for the Study of Liver Diseases and European Association for the Study of the Liver.^20^^-^^22^ The FIB-4 index is valuable for identifying high-risk patients, particularly in community screenings or primary care settings. Nevertheless, its applicability across different ages, body mass indices, and metabolic statuses remains controversial, and its sensitivity for monitoring disease progression is limited.^23^^-^^26^

Fibroblast growth factor 21 (FGF-21), a liver-derived hormone reflecting metabolic status, has gained attention in MASLD research due to its close association with lipid dysregulation, insulin resistance, and metabolic stress.^27^^-^^29^ Studies report elevated serum FGF-21 levels in patients with MASLD and MASH, likely reflecting hepatic responses to metabolic disturbances.^30^ This pattern has been observed across diverse metabolic contexts, including non-obese individuals living with human immunodeficiency virus infection and patients with obesity-associated MASH, suggesting the potential utility of FGF-21 as a serum biomarker.^31^^,^^32^

Although preliminary studies have explored the utility of FGF-21 in liver disease risk assessment, its performance in predicting MASLD fibrosis stages remains unclear and requires systematic validation. Compared with conventional scoring models, FGF-21, as a metabolic stress–related indicator, may provide additional information for fibrosis risk stratification, particularly in metabolically complex MASLD populations.

The current study evaluated serum FGF-21 levels and FIB-4 indices in patients with MASLD to assess their individual and combined predictive performance in fibrosis risk stratification. Given the lack of systematic research on the combined evaluation of FGF-21 and FIB-4, the current study characterized their potential utility in noninvasive MASLD screening and provided a basis for developing more effective early diagnostic strategies.

## Materials and Methods

### Study Subjects

This prospective cohort study enrolled patients with MASLD from The Second Affiliated Hospital of Harbin Medical University, a tertiary hospital, between January 2023 and December 2024. MASLD was diagnosed according to the 2020 international consensus criteria,^1^ requiring evidence of hepatic steatosis (imaging or biochemical abnormalities) in the absence of other liver diseases and the presence of at least 1 metabolic risk factor. The study was approved by the Ethics Committee of The Second Affiliated Hospital of Harbin Medical University (Approval No. 202101HB-6; approval date: January 16, 2021), and all participants provided written informed consent. No generative AI or large language model (LLM) tools were used in the preparation.

### Inclusion Criteria

Inclusion criteria were as follows: (1) patients aged 18-75 years; (2) patients meeting MASLD diagnostic criteria; (3) patients willing to participate and provide informed consent; and (4) patients with complete clinical data and eligible blood samples.

### Exclusion Criteria

Exclusion criteria were as follows: (1) Patients previously treated with FGF-21 or its analogs (e.g., agonists or antagonists); (2) patients with significant alcohol consumption (>30 g/day for men, >20 g/day for women) in the past 2 years; (3) patients with concurrent liver diseases (e.g., viral hepatitis, autoimmune liver disease, primary biliary cholangitis, or Wilson’s disease); (4) patients receiving long-term hepatotoxic medications (e.g., glucocorticoids, methotrexate, or amiodarone); (5) pregnant or lactating women; (6) patients with primary or metastatic liver tumors; and (7) patients with missing key data or noncompliant samples.

### General Data and Laboratory Tests

Demographic and anthropometric data were collected, including sex age, body mass index (BMI = weight (kg)/height^2^ (m^2^)), and waist circumference (WC).

Peripheral venous blood samples were obtained from all participants after an overnight fast. All tests were completed within 24 hours of sample collection in accordance with clinical laboratory quality control protocols.

*Whole-blood analysis (ethylenediaminetetraacetic acid dipotassium salt (EDTA-K_2_) anticoagulant, purple-top tubes)*: Two 2 mL tubes were collected per participant. One tube was used for complete blood count (including platelet count (PLT)), measured using a Beckman Coulter LH 780 automated hematology analyzer. The other tube was used for glycated hemoglobin (HbA1c) analysis, performed using a TOSOH G8 automated HbA1c analyzer with matched reagents (Tosoh Corporation, Tokyo, Japan).

Serum analysis (clot activator and gel separator tubes): A 5-mL blood sample was collected and allowed to clot at room temperature for 30 minutes. Serum was separated by centrifugation at 3000 rpm (~1500 × *g*) for 10 minutes and either analyzed immediately or stored at −80°C. Biochemical parameters (ALT, AST, gamma-glutamyl transferase (GGT), alkaline phosphatase (ALP), total bilirubin (Tbil), albumin, fasting plasma glucose (FPG), total cholesterol (TC), triglycerides (TG), high-density lipoprotein cholesterol (HDL-C), and low-density lipoprotein cholesterol (LDL-C), etc.) were measured using a Beckman Coulter AU5800 automated biochemistry analyzer (Beckman Coulter, Inc., Brea, CA, USA).

### Calculated Indices

HOMA-IR = [FINS (µIU/mL) × FPG (mmol/L)]/22.5 and FIB-4 = [age (years) × AST (U/L)]/[PLT (×10^9^/L) × √ALT (U/L)], where HOMA-IR is homeostasis model assessment of insulin resistance and FINS is fasting insulin.

### Measurement of Fibroblast Growth Factor 21 Levels

Blood samples were drawn from fasting participants via venipuncture using serum-separating tubes containing clot activators. Following collection, approximately 5 mL of whole blood was left to coagulate for 30 minutes at ambient temperature before centrifugation at 1500 × *g* (3000 rpm) for 10 minutes to isolate serum. The supernatant was aliquoted and stored at −80°C until further analysis. FGF-21 concentrations were determined using a standardized enzyme-linked immunosorbent assay kit (Elabscience, Wuuhan, China; catalog no. E-EL-H0074) following the manufacturer’s protocol. Briefly, 100 μL of each serum sample was analyzed in duplicate by trained technicians. Optical density was measured at 450 nm using a BioTek Synergy H1 microplate reader (BioTek Instruments, Inc., Winooski, VT, USA), and sample concentrations were calculated using a pre-established calibration curve.

### Histological Fibrosis Staging Criteria

All enrolled patients underwent histological liver evaluation following the NASH Clinical Research Network scoring system, with fibrosis staged on a 0-4 scale: F0 indicated no fibrosis, F1 represented mild perisinusoidal or portal fibrosis, F2 denoted moderate perisinusoidal and portal fibrosis, F3 indicated bridging fibrosis, and F4 represented cirrhosis.^33^ Liver biopsies were reviewed by experienced pathologists blinded to clinical and laboratory data to reduce assessment bias. For analysis, fibrosis stages were grouped as early-stage fibrosis (F0-F1) and significant fibrosis (F2-F4), which served as the primary outcome variable for both individual biomarker analysis and combined diagnostic model evaluation. The F2 threshold was chosen as the clinically relevant cutoff point because of its established prognostic significance in disease progression.

### Statistical Analysis

All statistical analyses were performed using R software version 4.4.3, (R Foundation for Statistical Computing; Vienna, Austria). Data import and cleaning were conducted using the tidyverse package (including dplyr and readr), model results were processed using broom, visualizations were generated with ggplot2, binary logistic regression modeling and prediction were performed using rms, string manipulation was handled using glue, and receiver operating characteristic (ROC) curve analysis was implemented using pROC (an open-source package for R and S+ to analyze and compare ROC curves). Continuous variables are presented as mean ± SD and compared between groups using independent samples *t*-tests. Categorical variables are expressed as counts (percentages) and compared using *χ*^2^ tests or Fisher’s exact test, with a 2-tailed *P* < .05 considered statistically significant.

Univariate logistic regression analysis was performed with significant fibrosis (F2-F4 vs. F0-F1) as the dependent variable, and candidate clinical variables were entered sequentially. Variables with *P* < .05 in univariate analysis were included in the multivariate logistic regression model. To prevent multicollinearity, component variables of calculated indices (e.g., HOMA-IR and FIB-4) were not included simultaneously. Continuous variables in the multivariate analysis were standardized using *z*-scores (per 1 SD unit) to allow comparison of effect sizes across variables.

ROC curve analysis was conducted using pROC, with the area under the curve (AUC) calculated and 95% CIs determined using the DeLong method. Optimal diagnostic thresholds were identified by maximizing Youden’s index (Youden’s index = sensitivity + specificity − 1). Diagnostic performance was evaluated under 2 strategies: specificity ≥70% and sensitivity ≥70%. Diagnostic performance metrics included sensitivity, specificity, false-positive rate (FPR = 1 − specificity), false-negative rate (FNR = 1 − sensitivity), Youden’s index, and *P *values. A combined model was developed using binary logistic regression, with fibrosis stage (F0-F1 vs. F2-F4) as the dependent variable and FGF-21 and FIB-4 as independent variables. Predicted probabilities from this model were used to generate ROC curves and calculate the diagnostic performance metrics as described above. Model calibration was assessed using bootstrap validation with 500 iterations. Calibration performance was evaluated using calibration plots and quantified using calibration slope, calibration intercept, and mean absolute error, with a slope near 1.0 and an intercept near 0 indicating optimal calibration.

## Results

### Study Population and Baseline Characteristics

A total of 125 patients with MASLD were enrolled, including 91 (72.8%) with F0-F1 fibrosis and 34 (27.2%) with F2-F4 fibrosis. Compared with the F0-F1 group, patients with F2-F4 fibrosis had significantly greater WC (93.82 ± 8.79 cm vs. 89.78 ± 8.68 cm, *P* = .023), higher HOMA-IR (4.10 ± 1.36 vs. 2.84 ± 1.09, *P* < .001), elevated TC (2.41 ± 0.50 mmol/L vs. 2.11 ± 0.47 mmol/L, *P* = .002), increased LDL-C (3.42 ± 0.70 mmol/L vs. 3.04 ± 0.61 mmol/L, *P* = .003), higher AST (47.55 ± 8.16 U/L vs. 36.88 ± 8.04 U/L, *P* < .001), elevated FINS (15.70 ± 5.57 μIU/mL vs. 12.65 ± 4.65 μIU/mL, *P* = .006), and a higher prevalence of hypertension (47.1% vs. 26.4%, *P* = .027). Additionally, patients with F2-F4 fibrosis had significantly lower HDL-C levels (1.00 ± 0.22 mmol/L vs. 1.11 ± 0.18 mmol/L, *P* = .005). No statistically significant differences were observed between the 2 groups in age, sex, BMI, systolic blood pressure, diastolic blood pressure, ALP, albumin, Tbil, FPG, HbA1c, TG, ALT, PLT, or prevalence of diabetes (all *P* > .05). Detailed data are presented in Table 1.

### Elevated Fibrosis-4 Index and Fibroblast Growth Factor 21 Levels in F2-F4 Metabolic Dysfunction–Associated Steatotic Liver Disease Patients

**Figure 1.**
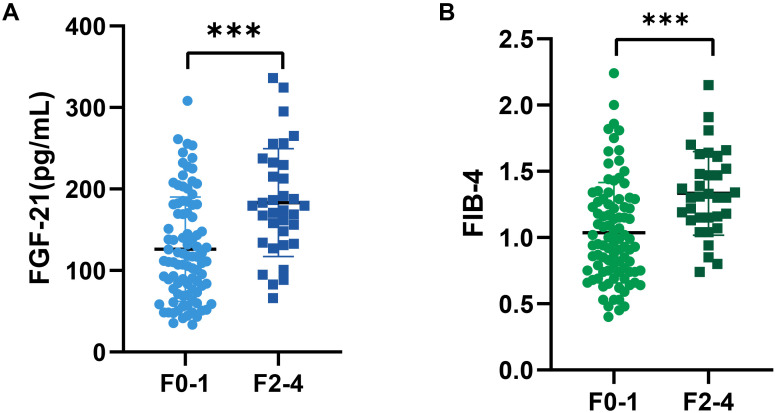
Comparison of FGF-21 and FIB-4 levels between patients with MASLD with different fibrosis stages (F0-F1 vs. F2-F4). (A) Serum FGF-21 levels were significantly higher in the F2-F4 group compared with the F0-F1 group. (B) FIB-4 index values were significantly elevated in the F2-F4 group compared with the F0-F1 group. Notes: Asterisks indicate statistical significance (* *P* < .05; ** *P* < .01; *** *P* < .001). FIB-4, fibrosis-4 index; FGF-21, fibroblast growth factor 21; MASLD, metabolic dysfunction–associated steatotic liver disease.

Compared with the F0-F1 group, patients with F2-F4 fibrosis had significantly higher serum FGF-21 levels [176.68 (137.76-225.88) vs. 111.83 (74.84-175.38) pg/mL, *P* < .001] and an elevated FIB-4 index [1.305 (1.150-1.510) vs. 0.97 (0.75-1.26), *P* < .001] (Table 2, Figure 1A, B). These findings indicate that both FGF-21 levels and FIB-4 index were consistently higher in the F2-F4 group compared with the F0-F1 group across different fibrosis stages.

### Univariate and Multivariate Logistic Regression Analysis

To avoid multicollinearity, original component variables of calculated indices (e.g., HOMA-IR, FIB-4) were not simultaneously included in the logistic regression analysis. In univariate logistic regression, factors significantly associated with significant liver fibrosis (F2-F4) analysis in patients with MASLD included WC [odds ratio (OR) = 1.055, 95% CI: 1.006-1.105, *P *= .026], GGT (OR = 1.033, 95% CI: 1.006-1.061, *P* = .016), HOMA-IR (OR = 1.989, 95% CI: 1.331-2.972, *P* < .001), TC (OR = 3.863, 95% CI: 1.591-9.379, *P* = .003), HDL-C (OR = 0.055, 95% CI: 0.007-0.455, *P* = .007), LDL-C (OR = 2.512, 95% CI: 1.327-4.757, *P* = .005), FIB-4 index (OR = 8.383, 95% CI: 2.635-26.667, *P* < .001), and serum FGF-21 levels (OR = 1.013, 95% CI: 1.006-1.019, *P* < .001). The results are presented in Table 3.

**Figure 2.**
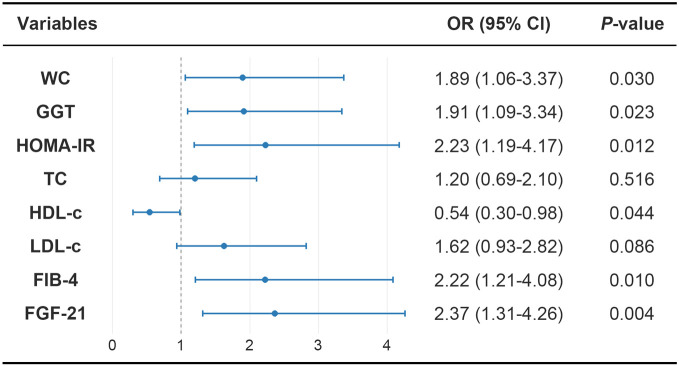
Forest plot of multivariate logistic regression analysis for significant liver fibrosis (F2-F4) in patients with MASLD. Continuous variables were standardized (per SD) to facilitate comparison of effect sizes. Only variables with *P* < .05 in univariate analysis (Table 3) were included in the multivariate model. FGF-21, fibroblast growth factor 21; FIB-4, fibrosis-4 index; GGT, γ-glutamyl transferase; HDL-C, high-density lipoprotein cholesterol; HOMA-IR, homeostasis model assessment of insulin resistance; LDL-C, low-density lipoprotein cholesterol; MASLD, metabolic dysfunction–associated steatotic liver disease; TC, total cholesterol; WC, waist circumference.

Variables with *P* < .05 in univariate analysis were included in the multivariate logistic regression model, with continuous variables standardized as *z*-scores (per 1 SD increment). Multivariate logistic regression analysis identified FIB-4 (OR = 2.22, 95% CI: 1.21-4.08, *P* = .010), FGF-21 (OR = 2.37, 95% CI: 1.31-4.26, *P* = .004), WC (O*R* = 1.89, 95% CI: 1.06-3.37, *P* = .030), GGT (O*R* = 1.91, 95% CI: 1.09-3.34, *P* = .023), and HOMA-IR (OR = 2.23, 95% CI: 1.19-4.17, *P* = .012) as independent risk factors, whereas HDL-C (OR = 0.54, 95% CI: 0.30-0.98, *P* = .044) was identified as a protective factor (Figure 2).

### Receiver Operating Characteristic Curve Analysis and Diagnostic Performance Evaluation

**Figure 3.**
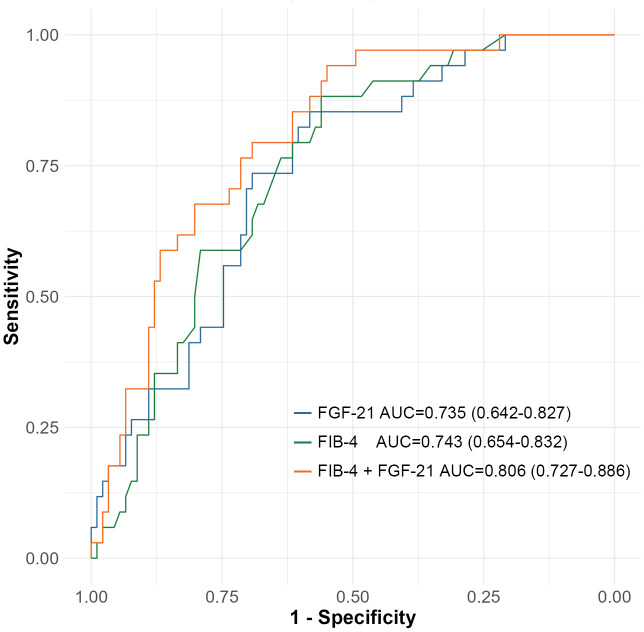
ROC curve analysis comparing FIB-4, FGF-21, and their combined model for detecting significant liver fibrosis (F2-F4) in patients with MASLD. AUC, area under the curve; FGF-21, fibroblast growth factor 21; FIB-4, fibrosis-4 index; MASLD, metabolic dysfunction–associated steatotic liver disease.

ROC curve analysis (Figure 3) demonstrated that the combined use of FIB-4 and FGF-21 yielded the highest diagnostic performance for significant liver fibrosis (F2-F4) in MASLD (AUC = 0.806, 95% CI: 0.727-0.886), outperforming FGF-21 alone (AUC = 0.735, 95% CI: 0.642-0.827) and FIB-4 alone (AUC = 0.743, 95% CI: 0.654-0.832).

Three cutoff strategies were evaluated to accommodate different clinical scenarios (Table 4). The Youden index-based cutoff maximized overall diagnostic efficiency, whereas alternative thresholds prioritized either high specificity (≥70%) for confirmatory testing or high sensitivity (≥70%) for screening purposes. Selection of optimal cutoff depends on the clinical context and resource availability.

Using the Youden index (primary strategy), the combined model achieved a sensitivity of 94.12% and specificity of 54.95% at a cutoff of 0.159 (Youden index = 0.491). Under the specificity ≥70% strategy (alternative 1), the model achieved a sensitivity of 82.35% and a specificity of 71.43% at a cutoff of 0.190. Under the sensitivity ≥70% strategy (alternative 2), the model (cutoff = 0.259) achieved a sensitivity of 70.59% and a specificity of 73.63%. Comparative performance across all 3 biomarkers and strategies is presented in Table 4.

### Model Calibration Assessment

**Figure 4.**
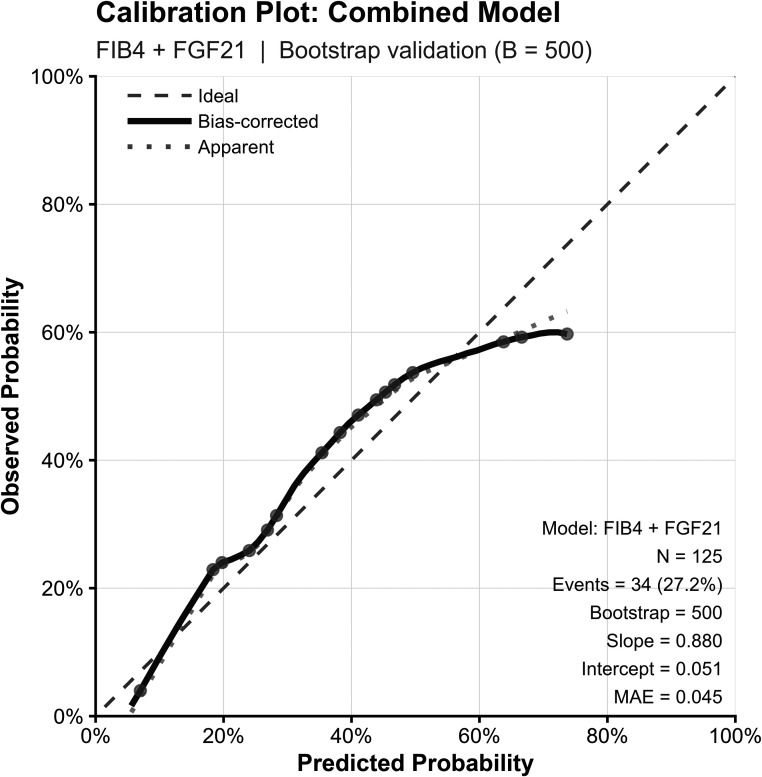
Calibration plot for the combined FIB-4/FGF-21 model. A calibration plot shows the agreement between predicted probabilities and observed frequencies of significant fibrosis (F2-F4). The diagonal dashed line represents perfect calibration, the solid line represents the bias-corrected calibration curve (bootstrap validation with 500 iterations), and the dotted line represents the apparent calibration. The model demonstrated good calibration with a calibration slope of 0.880, an intercept of 0.051, and a mean absolute error of 0.045. FIB-4, fibrosis-4 index; FGF-21, fibroblast growth factor 21.

Bootstrap validation with 500 iterations was performed to assess model calibration. The combined FIB-4/FGF-21 model demonstrated good calibration performance, with a calibration slope of 0.880, calibration intercept of 0.051, and a mean absolute error of 0.045. The calibration plot (Figure 4) showed close agreement between predicted and observed probabilities across the full range of risk, and the bias-corrected calibration curve closely approximated the ideal diagonal line, indicating minimal overfitting and reliable probability estimates for clinical application.

## Discussion

Previous studies have demonstrated that significant fibrosis (≥F2) in patients with MASLD is strongly associated with increased mortality and risk of liver transplantation.^34^ Therefore, clinical management should focus not only on identifying advanced fibrosis but also emphasize early detection of patients at stage F2 to enable timely intervention during the reversible phase of disease progression, thereby supporting secondary prevention. Although currently used noninvasive scoring models such as FIB-4 perform well in detecting advanced fibrosis, their ability to distinguish significant fibrosis (F2-F4) from early-stage fibrosis (F0-F1) remains suboptimal.^35^ FGF-21, a hepatokine closely linked to metabolic stress, is consistently elevated in patients with MASLD, suggesting its potential to provide additional predictive value beyond conventional models.^36^ Against this background, the current study stratified patients into F0-F1 and F2-F4 groups to investigate the association between metabolic indicators and fibrosis progression and further evaluated the performance of combined FGF-21 and FIB-4 for risk stratification

The results demonstrated significantly elevated FIB-4 index and FGF-21 levels in the F2-F4 group. FIB-4, a noninvasive scoring tool incorporating age, platelet count, ALT, and AST, correlates well with FibroScan-measured fibrosis stages and has been validated as a surrogate marker for significant fibrosis in MASLD.^37^ Similarly, FGF-21 shows increased hepatic expression and circulating levels in patients with MASLD/MASH, indicating an association with significant and advanced fibrosis.^36^ The observed progressive increase in FGF-21 with fibrosis stage is consistent with prior reports, supporting its potential as a sensitive biomarker for fibrosis progression in MASLD.

Comparative analysis showed significantly higher WC and LDL-C levels in the F2-F4 group. Large-scale cohort studies have confirmed that WC and related indices better demonstrate visceral fat distribution than BMI and are strongly associated with the development and prognosis of MASLD.^38^ Dyslipidemia, particularly elevated non-HDL-C to HDL-C ratios, has been identified as an independent risk factor for NAFLD/MASLD progression.^39^ Univariate logistic regression identified WC, GGT, HOMA-IR, HDL-C, TC, LDL-C, FIB-4, and FGF-21 as significant predictors of F2-F4 fibrosis. Multivariate analysis further confirmed FIB-4, FGF-21, WC, GGT, and HOMA-IR as independent risk factors, whereas HDL-C served as a protective factor. These findings suggest that progression to significant fibrosis involves not only traditional components of metabolic syndrome and liver injury but also markers of metabolic stress markers (FGF-21) and fibrosis burden (FIB-4).

The FGF-21/FIB-4 combination model is designed to complement, rather than replace, existing risk stratification algorithms for MASLD. Although imaging-based modalities such as FibroScan and MRE provide superior diagnostic performance, their high cost and limited availability restrict widespread use, particularly in resource-limited settings. In contrast, the proposed serum-based model utilizes routinely available laboratory parameters, making it accessible for initial screening in primary care and community healthcare settings. Recent evidence indicates that FGF-21 serves as a biomarker for metabolic dysfunction in MASLD and that incorporating metabolic parameters with fibrosis indices may improve risk assessment.^40^^,^^41^ This model can serve as a first-line screening tool to identify patients requiring confirmatory imaging or biopsy, thereby optimizing resource allocation and improving patient triage efficiency within existing diagnostic pathways.

The combined FIB-4/FGF-21 model demonstrated good discrimination for significant fibrosis (≥F2), with an AUC of 0.806. In metabolically high-risk MASLD/NAFLD cohorts, FIB-4 has generally demonstrated only moderate discrimination for advanced fibrosis, with AUROC values typically ranging from 0.73 to 0.77.^42^^-^^44^ Consistent with these reports, FIB-4 alone showed similar performance in the current cohort (AUC 0.743), whereas addition of FGF-21 improved overall discrimination (AUC 0.806). This improvement is biologically plausible because FGF-21, a hepatokine closely linked to metabolic stress and MASLD progression, may capture disease-relevant information not fully reflected by routine fibrosis indices. FGF-21 alone achieved discrimination comparable to FIB-4 (AUC 0.735 vs. 0.743), and the combined model further improved performance by integrating signals related to both hepatic injury and metabolic dysregulation. The selection of optimal cutoff values should be guided by clinical context and intended use. The current analysis evaluated 3 distinct strategies, each suited to different diagnostic scenarios. The Youden index–based cutoff (0.159) maximized overall diagnostic efficiency, with a sensitivity of 94.12% and a specificity 54.95%. This approach is suitable for initial screening in primary care settings where the primary objective is to identify all patients who may require further evaluation for significant fibrosis in MASLD. High sensitivity minimizes false-negative results, reducing the likelihood of missed cases of significant fibrosis, although this comes at the expense of lower specificity.

For settings requiring higher certainty before proceeding to costly confirmatory testing (e.g., FibroScan or liver biopsy), the specificity-oriented cutoff (71.43%) provides a balanced approach that reduces unnecessary referrals while maintaining acceptable sensitivity. Conversely, the sensitivity ≥70% threshold (cutoff 0.259, sensitivity 70.59%, and specificity 73.63%) provides the best balance of both metrics and may be optimal for specialist settings where accurate risk stratification is paramount. The availability of these alternative cutoff strategies enables clinicians to tailor the diagnostic decision-making according to their specific clinical context, resource availability, and characteristics of the target population.

The current study has several limitations. First, the relatively modest sample size may limit statistical precision. Second, validation was based on internal bootstrap resampling; although calibration indicated limited optimism, external validation is still needed. Third, this was a single-center cohort from a Chinese tertiary hospital, and applicability to other settings and populations requires confirmation. Fourth, longitudinal changes in biomarkers and fibrosis were not assessed. Future studies should focus on prospective external validation in diverse cohorts, comparison with established imaging methods, and evaluation of longitudinal predictive utility.

The current study confirms that both FGF-21 and FIB-4 serve as effective indicators for assessing liver fibrosis risk in patients with MASLD, with their combination demonstrating superior diagnostic performance compared with either marker alone. The integrated model significantly improves sensitivity while maintaining specificity, enabling timely identification of high-risk patients who may benefit from further intervention while reducing unnecessary invasive procedures such as liver biopsy in low-risk individuals. This approach offers a simple, scalable, noninvasive screening tool for MASLD-related fibrosis, supporting improved risk stratification and contributing to more precise and efficient clinical decision-making.

## Figures and Tables

**Table 1.** Baseline Characteristics of Patients with MASLD Stratified by Fibrosis Stage (F0-F1 vs. F2-F4)

**Variables**	**Total (n = 125)**	**F0-F1 (n = 91)**	**F2-F4 (n = 34)**	***P***
Age (years)	47.53 ± 10.32	47.24 ± 10.18	48.32 ± 10.78	.603
Gender (male, %)	71 (56.8)	54 (59.3)	17 (50.0)	.348
BMI (kg/m^2^)	27.04 ± 3.06	26.87 ± 2.90	27.51 ± 3.46	.439
WC (cm)	90.88 ± 8.86	89.78 ± 8.68	93.82 ± 8.79	.023
SBP (mmHg)	125.21 ± 9.35	125.27 ± 9.35	125.07 ± 9.48	.917
DBP (mmHg)	78.13 ± 8.22	78.39 ± 8.41	77.44 ± 7.79	.566
GGT (U/L)	46.40 ± 15.73	44.28 ± 14.27	52.06 ± 18.15	.013
ALP (U/L)	87.19 ± 13.53	87.18 ± 14.02	87.22 ± 12.31	.989
Albumin (g/L)	44.37 ± 3.05	44.43 ± 2.90	44.19 ± 3.47	.695
Tbil (μmol/L)	13.86 ± 3.67	13.77 ± 3.63	14.08 ± 3.84	.677
FPG (mmol/L)	5.67 ± 0.79	5.65 ± 0.77	5.75 ± 0.87	.515
HbA1c (%)	6.21 ± 0.64	6.18 ± 0.61	6.31 ± 0.70	.321
FINS (μIU/mL)	13.48 ± 5.08	12.65 ± 4.65	15.70 ± 5.57	.006
HOMA-IR	3.18 ± 1.29	2.84 ± 1.09	4.10 ± 1.36	<.001
TG (mmol/L)	4.95 ± 0.76	4.87 ± 0.75	5.16 ± 0.75	.061
TC (mmol/L)	2.19 ± 0.49	2.11 ± 0.47	2.41 ± 0.50	.002
HDL-C (mmol/L)	1.08 ± 0.20	1.11 ± 0.18	1.00 ± 0.22	.005
LDL-C (mmol/L)	3.14 ± 0.66	3.04 ± 0.61	3.42 ± 0.70	.003
AST (U/L)	39.78 ± 9.35	36.88 ± 8.04	47.55 ± 8.16	<.001
ALT (U/L)	53.38 ± 15.45	50.83 ± 12.29	60.20 ± 20.47	.016
PLT (×10^9^/L)	243.96 ± 50.42	246.83 ± 39.41	236.29 ± 72.35	.425
Diabetes (%)	30 (24.0)	18 (19.8)	12 (35.3)	.116
Hypertension (%)	40 (32.0)	24 (26.4)	16 (47.1)	.027
Fibrosis stage distribution				
F0 (%)	52 (41.6)			
F1 (%)	39 (31.2)			
F2 (%)	15 (12.0)			
F3 (%)	13 (10.4)			
F4 (%)	6 (4.8)			

Continuous variables are presented as mean ± SD and categorical variables as n (%). *P *values were calculated using independent *t*-test for continuous variables and *χ*^2^ test for categorical variables between F0-F1 and F2-F4 groups. *P* < .05 was considered statistically significant. HOMA-IR = [FINS (µIU/mL) × FPG (mmol/L)]/22.5.

ALP, alkaline phosphatase; ALT, alanine aminotransferase; AST, aspartate aminotransferase; BMI, body mass index; DBP, diastolic blood pressure; FINS, fasting insulin; FPG, fasting plasma glucose; GGT, γ-glutamyl transferase; HbA1c, hemoglobin A1c; HDL-C, high-density lipoprotein cholesterol; HOMA-IR, homeostasis model assessment of insulin resistance; LDL-C, low-density lipoprotein cholesterol; MASLD, metabolic dysfunction–associated steatotic liver disease; PLT, platelet count; SBP, systolic blood pressure; TC, total cholesterol; Tbil, total bilirubin; TG, triglycerides; WC, waist circumference.

**Table 2.** Comparison of FGF-21 and FIB-4 Between Fibrosis Stages (F0-F1 vs. F2-F4) in Patients with MASLD

**Variables**	**Total (n = 125)**	**F0-F1 (n = 91)**	**F2-F4 (n = 34)**	***P***
FIB-4 index	1.10 (0.82-1.34)	0.97 (0.75-1.26)	1.31 (1.15-1.51)	<.001
FGF-21 (pg/mL)	131.78 (87.06-185.56)	111.83 (74.84-175.38)	176.68 (137.76-225.88)	<.001

Data are presented as median (interquartile range). *P* values were calculated by the Mann–Whitney *U*-test (2-tailed). FIB-4 formula: [Age (years) × AST (U/L)]/[PLT (×10^9^/L) × √ALT (U/L)].

FIB-4, fibrosis-4 index; FGF-21, fibroblast growth factor 21; MASLD, metabolic dysfunction–associated steatotic liver disease.

**Table 3.** Univariate Logistic Regression for Factors Associated with F2-F4 Fibrosis in MASLD

**Variable**	**Univariate Analysis**		
	**OR**	**95% CI**	***P***
Age (years)	1.01	0.972-1.05	.600
Gender (male)	0.685	0.31-1.512	.349
BMI (kg/m^2^)	1.073	0.94-1.225	.295
WC (cm)	1.055	1.006-1.105	.026
SBP (mmHg)	0.998	0.956-1.041	.916
DBP (mmHg)	0.986	0.939-1.035	.563
GGT (U/L)	1.033	1.006-1.061	.016
ALP (U/L)	1.000	0.971-1.030	.989
Albumin (g/L)	0.974	0.856-1.109	.693
Tbil (μmol/L)	1.023	0.919-1.139	.675
HbA1c (%)	1.376	0.734-2.580	.319
HOMA-IR	1.989	1.331-2.972	<.001
TG (mmol/L)	1.663	0.97-2.851	.064
TC (mmol/L)	3.863	1.591-9.379	.003
HDL-C (mmol/L)	0.055	0.007-0.455	.007
LDL-C (mmol/L)	2.512	1.327-4.757	.005
FIB-4	8.383	2.635-26.667	<.001
FGF-21 (pg/mL)	1.013	1.006-1.019	<.001

OR for continuous variables (WC, GGT, HOMA-IR, TC, HDL-C, LDL-C, FIB-4, and FGF-21) are reported per 1 SD increase to allow comparison of effect sizes across variables with different scales. A *P* < .05 was considered statistically significant. HOMA-IR = [FINS (µIU/mL) × FPG (mmol/L)]/22.5; FIB-4 = [age (years) × AST (U/L)] / [PLT (×10^9^/L) × √ALT (U/L)].

ALP, alkaline phosphatase; ALT, alanine aminotransferase; AST, aspartate aminotransferase; BMI, body mass index; DBP, diastolic blood pressure; FGF-21, fibroblast growth factor 21; FIB-4, fibrosis-4 index; FINS, fasting insulin; GGT, γ-glutamyl transferase; HbA1c, hemoglobin A1c; HDL-C, high-density lipoprotein cholesterol; LDL-C, low-density lipoprotein cholesterol; MASLD, metabolic dysfunction–associated steatotic liver disease; OR, odds ratio; PLT, platelet count; SBP, systolic blood pressure; TC, total cholesterol; Tbil, total bilirubin; TG, triglycerides; WC, waist circumference.

**Table 4.** Diagnostic Performance of FIB-4, FGF-21, and Their Combination for Detecting F2-F4 Fibrosis

**Threshold Type**	**Model**	**Cutoff**	**Sensitivity (%)**	**Specificity (%)**	**FPR (%)**	**FNR (%)**	**Youden Index**	***P***
Youden index	FIB-4 + FGF-21	0.159	94.12	54.95	45.05	5.88	0.491	<.001
	FGF-21	126.505	85.29	58.24	41.76	14.71	0.435	<.001
	FIB-4	1.030	88.24	56.04	43.96	11.76	0.443	<.001
Specificity ≥70%	FIB-4 + FGF-21	0.231	76.47	71.43	28.57	23.53	0.479	<.001
	FGF-21	153.630	70.59	70.33	29.67	29.41	0.409	<.001
	FIB-4	1.295	58.82	79.12	20.88	41.18	0.379	<.001
Sensitivity ≥70%	FIB-4 + FGF-21	0.259	70.59	73.63	26.37	29.41	0.442	<.001
	FGF-21	153.630	70.59	70.33	29.67	29.41	0.409	<.001
	FIB-4	1.155	70.59	65.93	34.07	29.41	0.365	<.001

The primary cutoff was determined by the Youden index (sensitivity + specificity − 1). Alternative thresholds were selected to meet clinical criteria (specificity or sensitivity ≥70%). Predictive probabilities from the combined model were calculated using logistic regression with standardized predictors (per 1 SD increase). *P *values for comparisons of AUCs were calculated using the DeLong test.

AUC, area under the ROC curve; FGF-21, fibroblast growth factor 21; FIB-4, fibrosis-4 index; FNR, false-negative rate; FPR, false-positive rate.

## Data Availability

The data that support the findings of this study are available on request from the corresponding author.
